# Clinico-Dermoscopic Report of Molluscum Dermatitis: A Pearly Puzzle in Focus

**DOI:** 10.7759/cureus.75835

**Published:** 2024-12-16

**Authors:** Paari Karuvelam Jeyaseelan, Aravind Baskar Murthy, Murali Narasimhan, Ramachandran Ramakrishnan

**Affiliations:** 1 Dermatology, Venereology and Leprosy, SRM Medical College Hospital and Research Centre, Chennai, IND; 2 Dermatology, Venereology and Leprosy, Kauvery Hospital, Chennai, IND

**Keywords:** dermoscopy, halo eczema, molluscum contagiosum, molluscum dermatitis, pox virus

## Abstract

We report an 18-year-old male who presented with a two-month history of a lesion over his right forearm with a one-week history of sudden increase in size associated with pain. General and systemic examinations were normal. Dermatological examination revealed a single tender, well-defined, pearly white to erythematous, dome-shaped nodule of approximately 6mm x 5mm x 5mm with central umbilication and surrounding erythema. Dermoscopy revealed a central poly lobular white-yellow amorphous structure with a peripheral punctiform vascular pattern, collarette of scaling, and circumferential homogenous red area. Intracytoplasmic eosinophilic and basophilic inclusion bodies were found on the Tzanck smear. With the above clinical and dermoscopic findings, a diagnosis of molluscum contagiosum (MC) with molluscum dermatitis was made. Following curettage and removal of the contents of the lesion, the surrounding inflammation resolved completely within 20 days. This report emphasizes the fact that curettage of the primary MC lesion itself leads to the resolution of molluscum dermatitis, negating the need for additional topical steroid therapy. The unreported dermoscopic features of molluscum dermatitis in this report provide valuable insights into distinguishing molluscum dermatitis from other dermatological conditions with similar presentations.

## Introduction

Molluscum contagiosum (MC) is a common viral infection of keratinocytes with characteristic intracytoplasmic inclusions presenting with umbilicated papules with a translucent glossy appearance [1,2]. It is caused by the molluscum contagiosum pox virus (MCV) of which there are four major viral types - MCV-1, MCV-2, MCV-3, and MCV-4, the most prevalent serotype in the general population and human immunodeficiency virus (HIV)-infected individuals being MCV-1 and MCV-2 respectively [2-4]. Halo dermatitis, also known as halo eczema, is an eczematous reaction that usually develops around benign lesions like melanocytic nevi. Molluscum dermatitis refers to the halo eczema observed around MC lesions, leading to eventual regression of the primary lesion [3,5,6]. Here we report a clinico-dermoscopic report of MC with molluscum dermatitis, emphasizing the role of non-invasive investigations like dermoscopy in diagnosing the same.

## Case presentation

**Figure 1:**
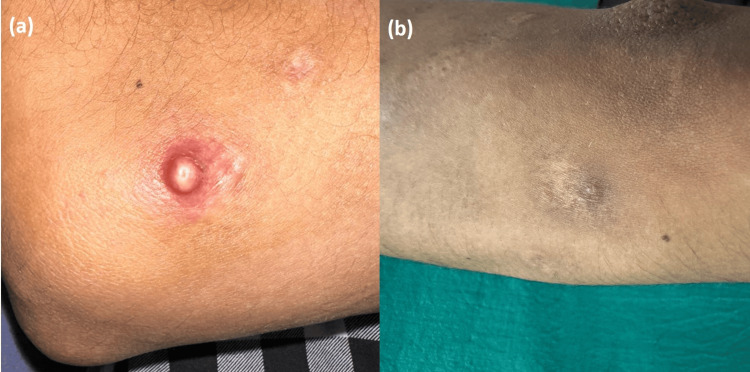
(a) A single well-defined erythematous dome-shaped nodule of size 1 cm x 1 cm with central umbilication, with surrounding erythema of 3 cm diameter over the lateral aspect of the right elbow (b) Follow-up image of the resolved lesion after 20 days

An 18-year-old male presented with a two-month history of a lesion over his right forearm and a one-week history of a sudden increase in the size of the lesion associated with pain. There was no history of any topical application. General and systemic examinations were normal. Dermatological examination revealed a single tender well-defined pearly white to erythematous, dome-shaped nodule of size approximately 1 cm x 1 cm with central umbilication, with surrounding erythema of diameter 3 cm and mild warmth over the lateral aspect of the right elbow (Figure 1).

**Figure 2:**
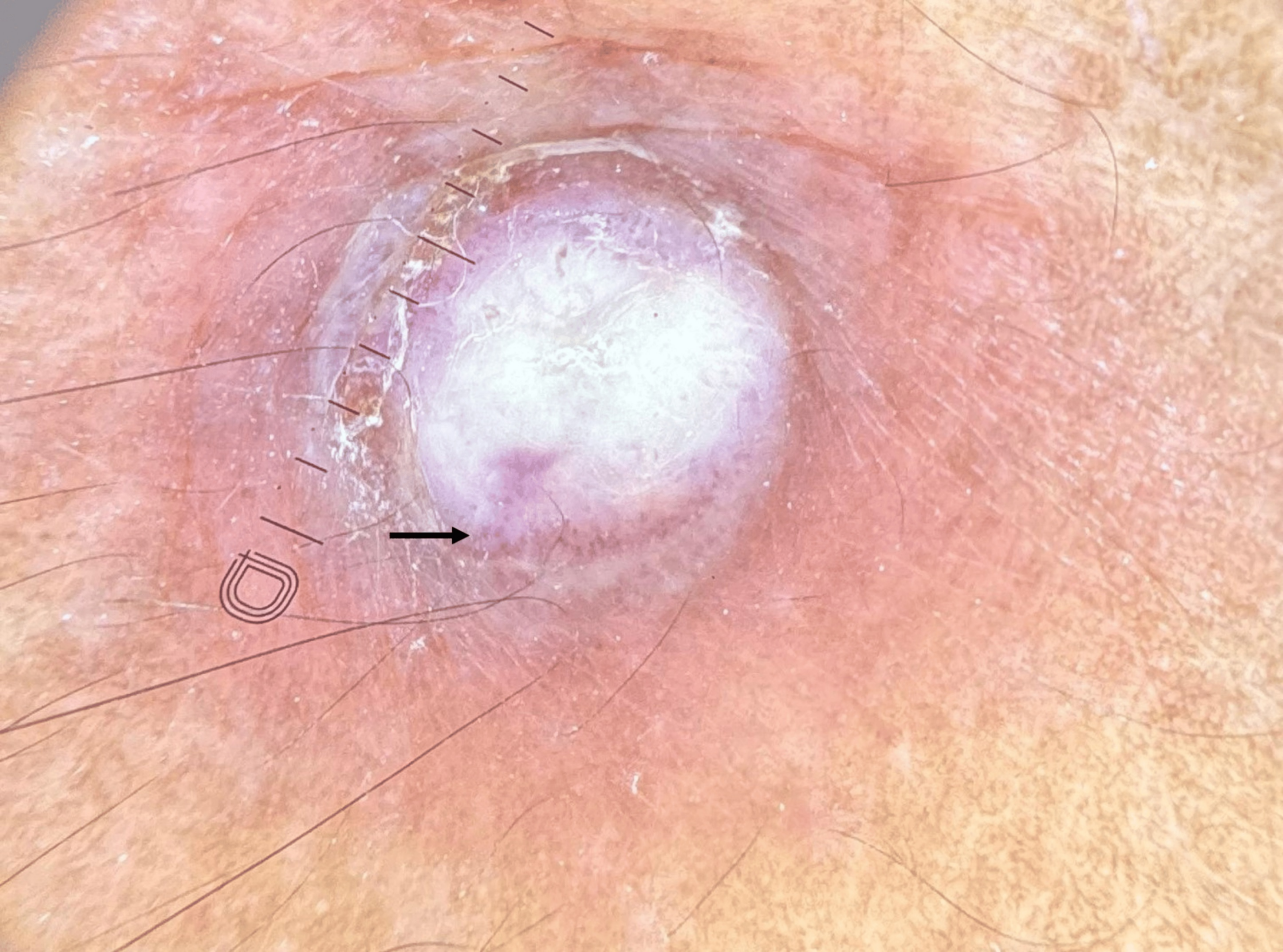
Dermoscopy (polarised mode, Dermlite DL 5, 10x magnification) of the lesion showing a central poly lobular white-yellow amorphous structure with a punctiform vascular pattern (black arrow), collarette of scaling, and circumferential homogenous red area.

Dermoscopy (polarised mode, 10x magnification; Dermlite DL 5, San Juan Capistrano, CA, USA) revealed a central poly lobular white-yellow amorphous structure with a punctiform vascular pattern, collarette of scaling, and circumferential homogenous red area (Figure 2).

**Figure 3:**
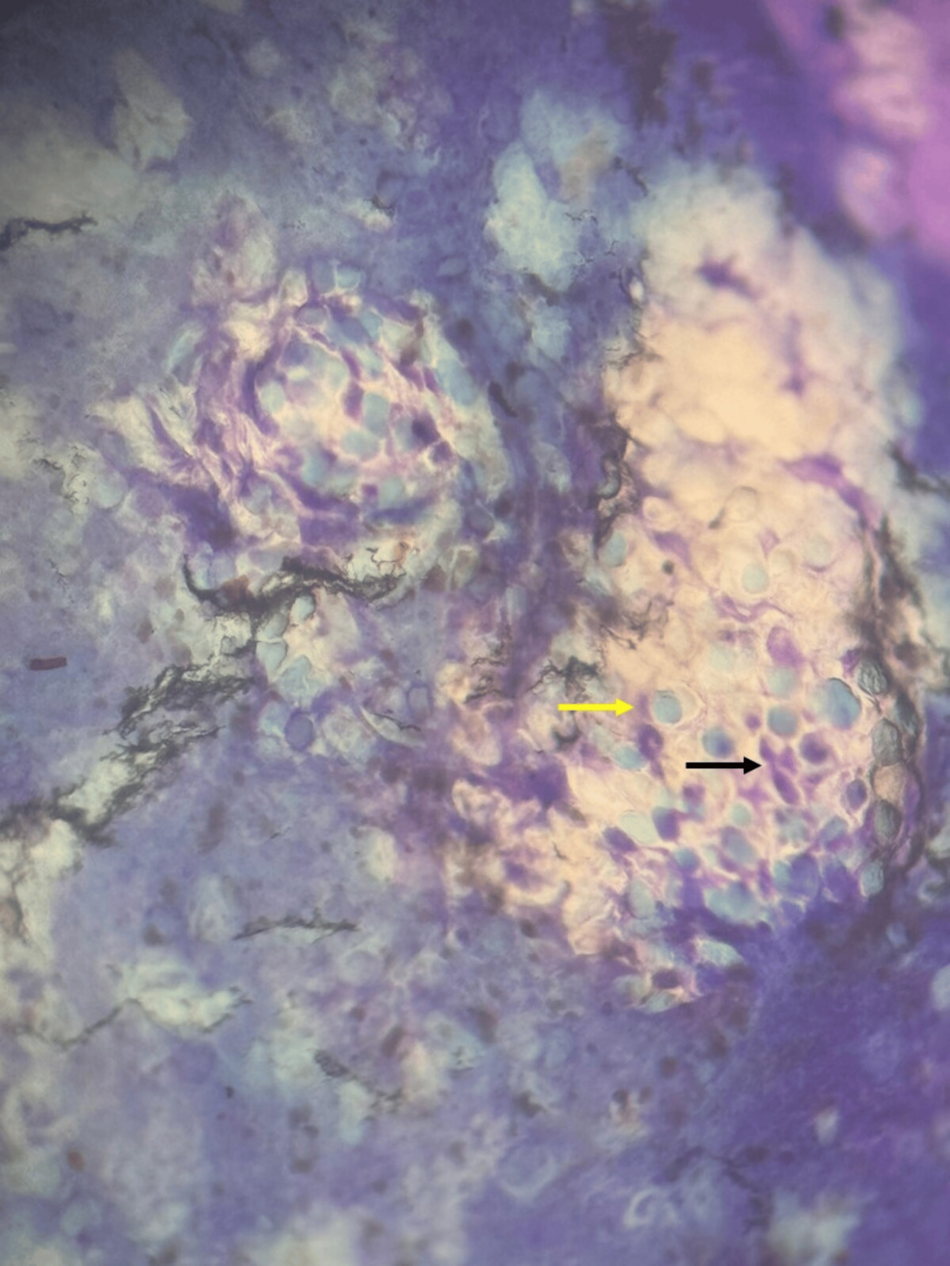
Tzanck smear of the contents of the lesion showing intracytoplasmic eosinophilic and basophilic inclusion bodies denoted by black and yellow arrows respectively (Henderson Paterson bodies).

Intracytoplasmic eosinophilic and basophilic inclusion bodies were found on the Tzanck smear of the contents of the lesion (Figure 3). Based on the clinical and dermoscopic findings, a diagnosis of molluscum contagiosum with molluscum dermatitis was made. Following curettage and removal of the contents of the lesion, the surrounding inflammation resolved completely within a period of 20 days.

## Discussion

MC infections have been reported across the world and are more frequently seen in children of two to five years of age and immunocompromised individuals with similar prevalence in males and females [2,3,7]. It occurs in nearly 7% of children with a prevalence of up to 18% in immunocompromised adults with HIV [3,7,8].

MCV may be transmitted by direct contact with infected skin, which can be sexual or through fomites such as towels and bathing sponges [3,9]. It may also further spread in the same individual through autoinoculation, called the pseudo-Koebner phenomenon [3,7]. The incubation period of MC infection ranges between two and six weeks [3]. Patients infected with MCV present firm rounded papules that are pearly white or skin-colored, with a shiny and umbilicated surface from 2 to 5 mm in size [3]. Rare presentations such as lesions larger than 1 cm in size, disseminated lesions, verrucous papules, and molluscum dermatitis have been observed [10].

The various cutaneous lesions associated with MC include molluscum dermatitis, Gionotti-Crosti syndrome-like reaction, erythema annulare centrifugum, erythema multiforme, id reaction, white halo, and folliculitis [11].

Halo dermatitis, also known as halo eczema, is a benign inflammatory eczematous eruption, usually observed around melanocytic nevi [6]. Halo dermatitis was first described by Meyerson in 1971, and hence also referred to as Meyerson's nevus or Meyerson's phenomenon [6]. MC lesions may also develop halo eczema, which is known as “molluscum dermatitis” [3,5]. Molluscum dermatitis, observed in 10% of MC patients, refers to the eczematous reaction around one or more MC lesions [12]. Molluscum dermatitis is triggered by manipulation of MC lesions leading to deposition of molluscum bodies in the dermis, which is followed by intense dermal inflammatory reaction leading to regression of lesions [12].

The MC virus alters the host immune response by encoding genes that are known to target human genes responsible for encoding major histocompatibility complex (MHC) class I molecules, interferons, interleukins, and chemokines [12]. Molluscum dermatitis comprises a composite inflammatory reaction consisting of predominantly effector T cells admixed with plasmacytoid dendritic cells, resembling interferon-dendritic cell (IFN-DC), and a unique type of CD123+ cell with an elongated and irregular nucleus [12]. Plasmacytoid dendritic cells release a large number of proinflammatory cytokines, attracting immune cells, and resulting in an inflammatory reaction [12]. Molluscum dermatitis is also attributed to localized sensitization to the elementary bodies of the MC virus or soluble products of its metabolism [13].

The average size of the lesions ranges from 3 to 10 cm. The reaction appears after an interval of one to 11 months [13,14]. The lesions are usually brownish-red, edematous, and associated with itching and scaling with reports of distant eczematous reactions [14]. Patients with atopic dermatitis are more prone to develop molluscum dermatitis [14,15]. The spontaneous resolution of MC lesions following the development of lesional inflammatory reaction is referred to by the acronym "Beginning of The End" (BOTE) sign, predicting the impending resolution of MC lesions [14,15]. Molluscum dermatitis is usually mistaken for secondary bacterial infection and unnecessarily treated with topical and systemic antibiotics. In a multicenter study assessing the prevalence of bacterial infection in acutely inflamed MC lesions, it was found that only 11.1% had skin-infecting organisms (Streptococcus pyogenes and Staphylococcus aureus) [16]. It was concluded in the study that, in lesions highly suggestive of bacterial infection like abscess or purulent discharge, it is advisable to do bacterial culture and sensitivity and initiate empiric treatment [16]. Id reaction or autoeczematization in MC with molluscum dermatitis has also been reported [17].

Molluscum dermatitis subsides shortly after or with the resolution of the involved MC lesion [13,18]. The differential diagnoses of molluscum dermatitis include secondary bacterial infection, allergic contact dermatitis, irritant contact dermatitis, and perilesional erythema annulare centrifugum [16,19,20]. Other conditions presenting with halo eczema are melanocytic nevus, seborrheic keratosis, dermatofibroma, keloid, lentigo benigna, insect bite, Behçet’s disease (under treatment with interferon α-2b), basal cell carcinoma and squamous cell carcinoma [21,22].

Histopathological examination (HPE) of MC lesions shows a lobulated crateriform lesion with acanthotic epidermis and eosinophilic to basophilic intracytoplasmic inclusion bodies referred to as molluscum bodies (Henderson Paterson bodies). HPE of molluscum dermatitis shows acanthosis, parakeratosis, spongiosis, dermal edema, and lymphohistiocytic infiltrates with occasional eosinophils in the dermis [23]. Dermoscopy of MC lesions shows a central yellowish-white structure corresponding to endophytic epidermal hyperplasia with intracytoplasmic inclusion bodies, surrounded by vascular structures and rosettes [1,24-26]. The various vascular patterns observed are crown, radial, and punctiform, the most common being the crown pattern [1,10,27]. Punctiform vascular pattern, though not exclusive to molluscum lesions, is most commonly associated with molluscum dermatitis [24,27]. Though dermoscopy of MC is well studied, there is a paucity of data in the literature on dermoscopy of molluscum dermatitis. The other investigations include enzyme-linked immunosorbent assay (ELISA) to look for IgG antibodies to MCV and electron microscopy, which shows characteristic brick-shaped poxvirus particles [28].

The various topical options for the treatment of MC include bichloroacetic acid, trichloroacetic acid, 10-20% potassium hydroxide, glycolic acid, salicylic acid, lactic acid, imiquimod, tretinoin, podophyllotoxin, cantharidin, sinecatechin, diphencyprone, cidofovir, and berdazimer. The intralesional therapies include interferon α, cidofovir, and candida antigen. The other therapeutic modalities include oral cimetidine, curettage, cryotherapy, and pulsed dye laser (PDL) [3,5,9,29]. Molluscum dermatitis resolves spontaneously in one to three weeks after the treatment of MC and thus does not require any specific treatment [13].

## Conclusions

The unreported dermoscopic features of molluscum dermatitis in this report provide valuable insights into distinguishing molluscum dermatitis from other dermatological conditions with similar presentations. This report also emphasizes the fact that curettage of the primary MC lesion itself leads to the resolution of molluscum dermatitis, negating the need for additional topical steroid therapy. Future studies could further explore dermoscopic variations across different patient populations of molluscum dermatitis, potentially enhancing diagnostic accuracy and therapeutic approaches.
